# The Book World of Medicine and Science

**Published:** 1897-09-11

**Authors:** 


### The Book World of Medicine and Science.

Digestion and Diet. With an Appendix on "The Opium Habit in India." By Sir William Roberts, M.D., F.R.S. Second edition. (London: Smith, Elder, and Co. 5s.)

Substantially, the second edition of this popular work by Sir William Roberts is the same as the first, which appeared some time ago; but it contains, in the form of an appendix, an article on "The Opium Habit of India," which first appeared as an annexure to the report of the Royal Commission published in 1895. Although one chapter is exclusively devoted to "Practical Hints on Dietetics," the value of the work in the hands of medical practitioners lies in the fact that every point elucidated and every theoretical question discussed has a practical significance for those who have the charge of the sick. "The profession" is continually brought into disrepute, and moral influence is lost, by the contradictory and chaotic instructions which are dispensed for the laity with dogmatic emphasis by various and sundry exponents of the science of medicine when any matter of dietetic treatment comes in question. To make order of this chaos, and to enunciate fundamental principles which shall introduce some sort of harmony into the rules and regulations of invalid dietary, has been the chief object of Sir William Roberts in collecting together under one cover the various lectures and papers for which from time to time he has been responsible. Moderation and common sense form the key-note for most of his teaching. For instance, in making suggestions for diet in cases where there are no "special indications to fulfil," his rule is to ask two questions before advising as to any particular article of food —namely, do you like it? and does it agree with you? If the answer be in the affirmative, "there is no intelligible reason why the use of that article should not be sanctioned." "The indications of the palate are of great importance in the regulation of the diet." If a few of us were guided by general principles of this broad order, as a body we should make far fewer mistakes, and less often lose the confidence of our patient by the grossest of contradictions. In the preparation of artificial foods Sir William Roberts is prolific in "tips," if one may use the expression. For instance, the ordinary peptonised food inflicted on many unfortunate typhoid patients is distinctly disagreeable and unpalatably bitter. It can be made particularly nice, as he who reads may observe on page 194 and onwards. The reading of "Digestion and Diet" cannot fail to be profitable investment for all practitioners. Errors will be avoided, and the chances of success in treatment increasingly enhanced.

Diphtheria and Anti-toxin. By Nestor Tirard, M.D. (London: Longmans, Green, and Co. 1897. 7s. 6d.)

We have read this little summary of diphtheria and its treatment with much pleasure. In undertaking a task of this kind the writer is almost necessarily hampered in his work by natural instincts to prejudice the cause which he espouses by quoting long arrays of statistics in support of his own particular views. In this volume we are pleased to notice a fair-minded and dispassionate review of the subject and a most commendable absence of statistical tables. That Dr. Tirard is a firm believer in the anti-toxin treatment is of course obvious from the very first, but it could scarcely be otherwise in view of the success which this line of treatment has met with in his own hands. But to the portion which deals with this particular subject he has devoted no disproportionate allowance of space. The whole subject of diphtheria is fairly dealt with; to the history, the etiology, the pathology, and the symptomatology is apportioned a legitimate and well-balanced degree of importance. In a couple of hours the student can make himself master of the contents, and, we cannot help thinking, with considerable advantage to himself. In a work so estimable it is perhaps a refinement of critical unkindness to point out two inaccuracies of expression which we have noticed, but on page 43 Dr. Tirard, in speaking of diphtheritic paralysis following on the anti-toxin treatment, says: "And it may here be noted that the relative increase of diphtheritic paralysis which has been stated to follow the anti-toxin treatment may be probably explained by the decrease in the mortality produced by the anti-toxin. As a larger number of patients survive a larger number remain to be attacked by diphtheritic sequelæ." This increase is absolute rather than relative. And, again, on page 17 the author speaks of albumen existing in urine to the extent of 30 per cent. He means by this, of course, what is usually called one-third albumen, and which is itself a loose and inaccurate expression for saying that, on boiling, the deposit formed is about one-third of the entire amount, representing roughly 1·7 per cent. albumen. These small inaccuracies, which we noticed, and which are the only ones we can find, show the interest we have taken in the reading, and we cannot help thinking that there must be many who will take as much pleasure in the perusal as we have done ourselves.

Convergent Strabismus and its Treatment. An Essay by Edwin Holthouse, M.A., F.R.C.S., Surgeon to the Western Ophthalmic Hospital. (London: J. and A. Churchill. 1897. Pp. 177. Price 6s.)

This book is the outcome of a well-planned and painstaking endeavour to clear up some of the difficult points in connection with convergent strabismus, which, we agree with the author, is far from being "a threshed-out subject." We regret that, seeing how common an affection convergent strabismus is, Mr. Holthouse did not wait until he had collected a larger number than 144 cases before drawing up his tables. His conclusions, if drawn from several hundred cases, would have carried far greater weight, especially where he deals with the exceptional forms of this affection. Some of the points he brings out are of considerable interest. He shows, for instance, that in 30 per cent. of his cases an hereditary tendency to squint was clearly present, and infers that inherited predisposition has a potent influence in determining its onset; that whilst ametropia was present in all the fixing eyes, its more complex forms were found in a higher ratio among the deviating eyes; that the higher the ametropia of the fixing eye, the greater is likely to be the deviation of the other. He doubts if the ordinary belief, that convergent strabismus is rarely found associated with a high degree of hypermetropia, is correct, as he found it to be comparatively frequently present in his series of cases. In connection with the vexed question as to whether amblyopia in squinting eyes is a congenital defect or the result of disease, he shows that the worst degrees of amblyopia in deviating eyes is chiefly to be found among such cases as show marked depreciation of vision in the fixing eye. The better the vision in the latter the less likely is that of the former to be hopelessly bad. Also, that the sight of the squinting eyes was not made worse by the longer duration of the squint. In the matter of treatment we should take exception to his statement, "that not until the fourth year has been reached can an ordinary child be expected to tolerate spectacles, or at any rate to wear them in such a way as to derive material benefit from them." Many children at three, and some as young as two, will wear glasses and appreciate them, crying for them when they are not put on. The book is clearly written, well printed, and will fully repay perusal by all those interested in ophthalmology.

